# Correlation between bony structures of the posterior cranial fossa and the occurrence of hemifacial spasm

**DOI:** 10.3389/fneur.2024.1418449

**Published:** 2024-07-03

**Authors:** Tianjin Tang, Wenqiang Yang, Qi Wang, Yanbing Yu, Li Zhang

**Affiliations:** ^1^Department of Neurosurgery, Xuzhou No.1 Peoples Hospital, Xuzhou, China; ^2^Department of Neurosurgery, China-Japan Friendship Hospital, Beijing, China

**Keywords:** hemifacial spasm, multiplanar reconstruction, posterior cranial fossa, skull, length

## Abstract

**Objective:**

To quantitatively study the measurement data related to the bony posterior cranial fossa and explore the correlation between bony posterior cranial fossa morphology and the occurrence of hemifacial spasm.

**Methods:**

A total of 50 patients with hemifacial spasm who attended the Department of Neurosurgery of China-Japan Friendship Hospital from October 2021 to February 2022 were included, and 60 patients with minor head trauma excluding skull fracture and intracranial abnormalities were included as controls. Cranial multilayer spiral CTs (MSCTs) were performed in both groups, and multiplanar reconstruction (MPR) was used as a postprocessing method to measure data related to the posterior cranial fossa in both groups.

**Results:**

Compared with the control group, the anteroposterior diameter (labeled AB) and the height (labeled BE) of the bony posterior cranial fossa, the anteroposterior diameter of the foramen magnum (labeled BC), the length of the clivus (labeled AB), and the length of the posterior occipital (labeled CD) in the HFS group were all reduced, and the differences were statistically significant. BE is positively correlated with AB and CD, with a stronger correlation observed between BE and AB (*r* = 0.487, *p* < 0.01). AB is negatively correlated with AD (*r* = −0.473, *p* < 0.01). The remaining correlations between the data were not statistically significant. There was no overlap in the 95% confidence interval for any of the measurements between the hemifacial spasm group and the control group.

**Conclusion:**

There is a correlation between the posterior cranial fossa and hemifacial spasm.

## Introduction

Hemifacial spasm (HFS) is a common clinical cranial nerve disease that clinically manifests as episodic, recurrent, involuntary tics of facial expression muscles in the area of facial nerve innervation. The disease progresses slowly, the duration of the disease is long, and severe symptoms have a huge impact on the patient’s life and work (1, 2). The vast majority of HFS cases are caused by vascular compression in the root exit zone (REZ) of the facial nerve, and microvascular decompression (MVD) is the only effective way to cure the disease by relieving the compression of the REZ blood vessels of the facial nerve (3–6). However, why HFS occurs in some people, how vascular compression nerves are produced, and whether there are anatomical specificities associated with the disease are unclear. In studies on the pathogenesis of HFS, no studies have been reported on the correlation between posterior fossa-related bone data and the pathogenesis of HFS in HFS patients. Therefore, we designed this case-control study to perform multislice hyperlux CT (MSCT) examination of the brain in HFS and control patients using multiple plane reconstruction (MPR) as a postprocessing method, quantitatively studying the relevant measurement data of the bony posterior fossa, and exploring the correlation between posterior fossa bony abnormalities and the incidence of HFS to further deepen the understanding of the etiology of HFS.

## Materials and methods

### Patient population

Fifty patients with HFS diagnosed in the neurosurgery department of China-Japan Friendship Hospital from October 2021 to February 2022 were selected by the random number table method, and patients with intracranial space-occupying lesions, vascular malformations, hydrocephalus and other secondary conditions were excluded. Because MSCT examination is radioactive, based on ethical considerations, the control group included 60 patients with mild head trauma who were admitted to China-Japan Friendship Hospital at the same time, excluding patients with skull fracture and intracranial abnormalities and excluding patients with HFS and brain surgery history. There were 30 males and 20 females in the HFS group, with an average age of 49.04 years. There were 36 males and 24 females in the control group, with an average age of 48.51 years. There was no significant difference in age or sex between the two groups. The study was designed with the approval of the Ethics Committee, and all subjects were informed of the nature of the study and signed an informed consent form.

### Imaging examination and measurement methods

**Figure 1:**
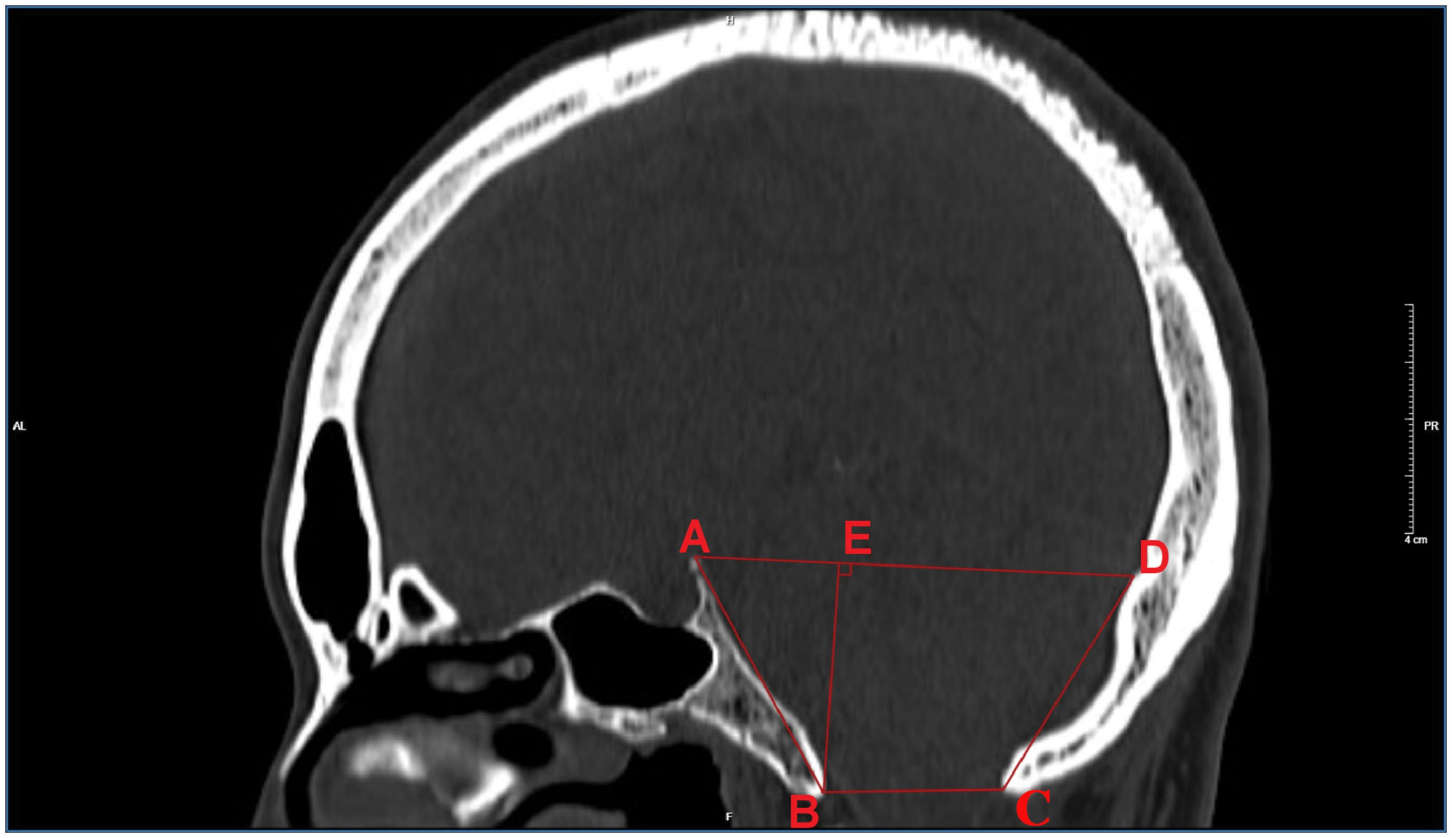
Schematic diagram of measuring method.

Philips Health care 256-slice spiral CT examinations were performed, and the standard optimized skull scanning plan was applied. The current was 250 mA, the voltage was 120 kV, the spiral scanning layer thickness was 2 mm, the interval was 2 mm, and the pitch was 10 mm. Reconstruction parameters: the layer thickness of the standard algorithm was 1.0 mm, and the reconstruction interval was 0.6 mm. The scanning range included the entire skull from the lower edge of the first cervical vertebra. The scanned data were reconstructed by the bone algorithm to obtain the original axial images, which were then transmitted to the image postprocessing workstation for the reconstruction of the sagittal image by MPR, with a window width/window position of 1,500 HU/400 HU. As shown in Figure 1, items measured on sagittal images with posterior fossa osseous data included: (1) the length of the clivus (marked AB), which was the distance from the vertex of the dorsum sellae to the bony front of the anterior lip of the foramen magnum; (2) the anterior-posterior diameter (marked as BC), which was the distance between the anterior and posterior bony edges of the lip of the foramen magnum; and (3) the length of the posterior occipital (marked as CD), which was the distance between the bony posterior edge of the foramen magnum and the intraoccipital tuberosity. (4) The anterior-posterior diameter (labeled AD) was the distance from the dorsum sellae to the intraoccipital tuberosity. (5) The height (labeled BE) was the minimum distance between the line from the sellar tubercle to the intraoccipital tuberosity and the line from the anterior to the posterior lip of the foramen magnum.

### Statistical analyses

SPSS23.0 statistical software was used for statistical analysis. All measurement data were first subjected to the Shapiro–Wilk test for normality, and the results indicated that the data met the assumption of normal distribution at the significance level of *α* = 0.05 (*p* > 0.05). The enumeration data were subjected to *χ*2 tests, and the measurement results of measurement data were all expressed as $\bar{x}\pm s$ . Independent sample *t* tests were used for intergroup comparisons to calculate the Pearson correlation coefficient (*r*). The 95% confidence intervals were calculated for both sets of measurements. *p* < 0.05 was considered statistically significant.

## Results

**Figure 2:**
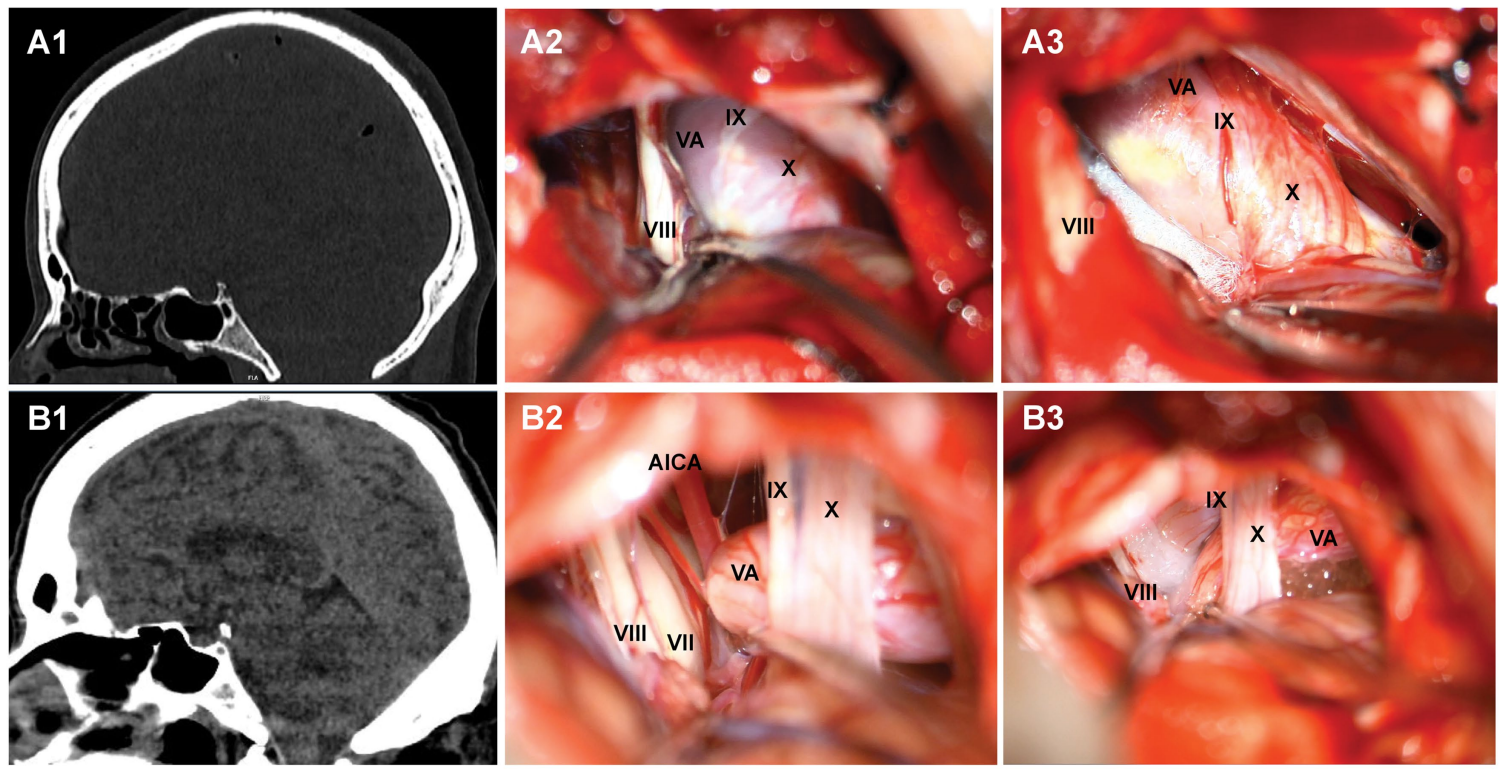
Two typical cases of hemifacial spasm. **A1, B1**: MPR post-processing after multi-slice spiral CT examination of the brain indicates that the posterior cranial fossa is narrow; **A2, B2**: exploration of the root of facial nerve in the cerebellopontine angle region revealed the compression of responsible vessels; **A3, B3**: placing padding cotton to separate the responsible blood vessels.

The comparison of the measurement results of the bony posterior fossa between the HFS group and the control group is shown in Table 1. The results showed that, compared with the control group, the measured values of AB, BC, CD, AD and BE in the posterior fossa of patients in the HFS group were decreased, and the differences were statistically significant. The calculation results of Pearson correlation coefficient (*r*) among the data (Table 2) indicate that BE is positively correlated with AB and CD, with a stronger correlation observed between BE and AB (*r* = 0.487, *p* < 0.01). AB is negatively correlated with AD (*r* = −0.473, *p* < 0.01). The remaining correlations between the data were not statistically significant. The 95% confidence intervals showed no overlap between the two groups (Table 3). Our results suggest that the size reduction and morphological abnormalities of the posterior fossa are associated with the development of HFS. Figure 2 shows two typical cases in the HFS group.

**Table 1:** Comparison of MSCT three-dimensional reconstruction measurement results between the control group and the hemifacial spasm group $\left(\bar{x}\pm s\right)\text{.}$

Variable (mm)	Control (*n* = 60)	HFS (*n* = 50)	*T* value	*p*-value
AB	47.49 ± 2.71	44.29 ± 5.48	3.977	<0.001
BC	35.43 ± 2.57	32.99 ± 3.20	4.425	<0.001
CD	46.15 ± 5.09	42.62 ± 5.15	3.597	<0.001
AD	83.81 ± 5.17	77.69 ± 9.49	4.292	<0.001
BE	40.39 ± 2.73	36.94 ± 3.15	6.172	<0.001

**Table 2:** The calculation results of Pearson correlation coefficient (*r*) among the hemifacial spasm group.

Variable (mm)	AB	BC	CD	AD	BE
AB	1				
BC	−0.069	1			
CD	−0.029	−0.150	1		
AD	−0.473^**^	0.185	0.189	1	
BE	0.487^**^	0.015	0.318^*^	−0.118	1

*p < 0.05; **p < 0.01.

**Table 3:** 95% CIs for measurements of bony posterior fossa in the control and hemifacial spasm groups.

Variable (mm)	Control (*n* = 60)	HFS (*n* = 50)
AB	46.79–49.19	42.74–45.85
BC	34.77–36.10	32.09–33.91
CD	44.83–47.46	41.16–44.09
AD	82.47–85.15	74.99–80.39
BE	39.69–41.10	36.04–37.83

## Discussion

The incidence of HFS is related to a variety of factors. Jannetta et al. have shown that with age, tortuous sclerosis of the intracranial artery, coupled with brain tissue atrophy, increases the probability of contact between the facial nerve REZ and adjacent blood vessels (6, 7). Other studies have shown that posterior fossa developmental malformations and volume reduction lead to congestion of the posterior fossa contents, increase the chance of vascular compression of the facial nerve REZ, and thickening of arachnoid adhesions to fix blood vessels to facial nerves or limit the elasticity and mobility of blood vessels, all of which contribute to the continuous compression of nerves by blood vessels (7–9). There have been case reports that patients with cerebellar tonsillar hernia and HFS who undergo simple posterior cranial decompression with the suboccipital median approach without facial nerve MVD not only alleviate the symptoms of cerebellar tonsillar hernia but also improve the symptoms of HFS (10). Concomitant HFS can be observed in some cases, such as in cases of cerebellar hematoma, posterior fossa tumor, hydrocephalus, and cerebellar tonsillar herniation, suggesting that a relatively small posterior fossa volume may be a risk factor for the development of HFS (11–13). Pontine cerebellar angle area tumors accounted for 0.8% of the cases of secondary HFS. In addition to the tumor directly compressing the facial nerve, there are also some tumors far away from the facial nerve REZ. The cause of the pathogenesis is simple vascular compression, and the mechanism may be due to the presence of tumors resulting in a relatively narrow posterior cranial space, increasing the probability of blood vessels contacting the facial nerve REZ (14).

The narrow space of the posterior cranial socket leads to crowding of the contents of the pontine cerebellar corner pool, which increases the chance of contact between blood vessels and the facial nerve REZ, and the facial nerve REZ is subjected to long-term compression of blood vessels and demyelinating changes, thereby transmitting signal impulses between nerve fibers and short circuiting, resulting in facial muscle twitching, so theoretically, people with a small posterior fossa space are more likely to develop HFS (8, 15). Literature reports show that women have a more crowded posterior fossa than men, and the probability of HFS in women is higher than that in men. There are also differences in the morphology and structure of the posterior fossa between different ethnic groups, and the incidence of HFS also varies (1).

Based on the above reasons, studying the relationship between posterior fossa volume and bony abnormalities and the pathogenesis of HFS is of great value for further understanding the etiology of HFS. In previous studies, there were some indicators to evaluate the morphology of the posterior fossa. On the one hand, due to the limitations of imaging technology at that time, the accuracy of the data was not satisfactory. On the other hand, some indicators require external measurement software, which lacks operability and simplicity. In this study, MSCT 3D reconstruction technology was used to measure the relevant data of the bony posterior fossa in the HFS group and control group, including the anteroposterior diameter of the bony posterior fossa, height of the bony posterior fossa, anteroposterior diameter of the foramen magnum, slope length, and posterior occipital length, which could basically reflect the bony structure morphology of the posterior fossa. Our results show that the measurement results of MSCT reconstruction of the bony posterior fossa are an important reference for the study of the etiology of HFS. Measuring the relevant data of the posterior fossa can provide a certain basis for clinical interpretation of the onset of hemifacial spasm. Posterior fossa size reduction and morphological abnormalities are associated with the onset of HFS.

Improvement of the accuracy of the results of this study requires analysis of larger samples at a later stage. In this study, MSCT scans were followed by only bone algorithm reconstruction, and soft tissue reconstruction was ignored, which could be supplemented in later studies. Prior to surgery, no vascular-related examinations were conducted on the patients. In subsequent research, we intend to emphasize the study of the relationship between vascular tortuosity morphology and hemifacial spasm, while also exploring whether morphological parameters are associated with the incidence of postoperative complications and the postoperative efficacy. Our results show that developmental abnormalities of the slope in HFS patients are more pronounced than other parts of the posterior fossa. However, no further and more comprehensive analysis of such abnormalities has been carried out, and further research on developmental abnormalities of the slope and sphenoid bone in HFS patients needs to be further improved.

## Data availability statement

The datasets presented in this study can be found in online repositories. The names of the repository/repositories and accession number(s) can be found in the article/supplementary material.

## Ethics statement

The studies involving humans were approved by China-Japan Friendship Hospital. Written informed consent for participation in this study was provided by the participants’ legal guardians/next of kin. Written informed consent was obtained from the individual(s), and minor(s)’ legal guardian/next of kin, for the publication of any potentially identifiable images or data included in this article. The animal studies were approved by China-Japan Friendship Hospital. Written informed consent was obtained from the owners for the participation of their animals in this study. Both studies were conducted in accordance with the local legislation and institutional requirements.

## Author contributions

TT: Writing – original draft, Writing – review & editing. WY: Data curation, Writing – review & editing. QW: Data curation, Formal analysis, Writing – review & editing. YY: Supervision, Writing – review & editing. LZ: Supervision, Writing – review & editing.
